# SGK1-dependent stimulation of vascular smooth muscle cell osteo-/chondrogenic transdifferentiation by interleukin-18

**DOI:** 10.1007/s00424-019-02256-5

**Published:** 2019-01-31

**Authors:** Nadeshda Schelski, Trang T. D. Luong, Florian Lang, Burkert Pieske, Jakob Voelkl, Ioana Alesutan

**Affiliations:** 1 Department of Internal Medicine and Cardiology, Charité – Universitätsmedizin Berlin, Campus Virchow-Klinikum, Augustenburgerplatz 1, 13353 Berlin, Germany; 2 Department of Physiology I, Eberhard-Karls University, Wilhelmstr. 56, 72076 Tübingen, Germany; 3 Berlin Institute of Health (BIH), Anna-Louisa-Karsch 2, 10178 Berlin, Germany; 4 Department of Internal Medicine and Cardiology, German Heart Institute Berlin, Augustenburger Platz 1, 13353 Berlin, Germany; 5 DZHK (German Centre for Cardiovascular Research), partner site Berlin, Hessische Str. 3-4, 10115 Berlin, Germany; 6 Institute for Physiology, Johannes Kepler University Linz, Altenberger Strasse 69, 4040 Linz, Austria

**Keywords:** SGK1, interleukin-18, PI3K, Vascular calcification, Osteo-/chondrogenic signaling, Vascular smooth muscle cells

## Abstract

The serum- and glucocorticoid-inducible kinase 1 (SGK1) is a key regulator of osteo-/chondrogenic transdifferentiation and subsequent calcification of vascular smooth muscle cells (VSMCs). The phenotypical transdifferentiation of VSMCs is associated with increased interleukin-18 (IL-18) levels and generalized inflammation. Therefore, the present study investigated the possible involvement of SGK1 in IL-18-induced vascular calcification. Experiments were performed in primary human aortic smooth muscle cells (HAoSMCs) treated with recombinant human IL-18 protein in control or high phosphate conditions and following SGK1 knockdown by siRNA or pharmacological inhibition of SGK1, PI3K, and PDK1. As a result, IL-18 treatment increased *SGK1* mRNA and protein expression in HAoSMCs. IL-18 upregulated *SGK1* mRNA expression in a dose-dependent manner. This effect was paralleled by upregulation of the mRNA expression of *MSX2* and *CBFA1*, osteogenic transcription factors, and of tissue-nonspecific alkaline phosphatase (*ALPL*), an osteogenic enzyme, as markers of increased osteo-/chondrogenic transdifferentiation. Phosphate treatment increased *SGK1* and osteogenic markers mRNA expression as well as ALPL activity and induced calcification of HAoSMCs, all effects significantly augmented by additional treatment with IL-18. Conversely, silencing of SGK1 or cotreatment with the SGK1 inhibitor EMD638683 blunted the effects of IL-18 on osteo-/chondrogenic transdifferentiation and calcification of HAoSMCs. The procalcific effects of IL-18 were similarly suppressed in the presence of PI3K or PDK1 inhibitors. In conclusion, SGK1 expression is upregulated by IL-18 in VSMCs and SGK1 participates in the intracellular signaling of IL-18-induced osteo-/chondrogenic transdifferentiation of VSMCs. Thus, SGK1 may serve as therapeutic target to limit the progression of medial vascular calcification during vascular inflammation.

## Introduction

Medial vascular calcification is frequently observed in aging, diabetes mellitus, atherosclerosis, and most extensively in chronic kidney disease (CKD) [32, 44]. In large arteries, vascular calcification may lead to increased stiffness, elevated pulse pressure and, thus, to cardiac hypertrophy and impaired coronary perfusion [18, 20]. Accordingly, vascular calcification is associated with an increased risk for cardiovascular events and cardiovascular and all-cause morbidity and mortality in CKD [35, 41, 44].

Vascular calcification is an active pathological process, promoted mainly by osteo-/chondrogenic transdifferentiation of vascular smooth muscle cells (VSMCs) [18, 28]. In CKD, various pathological factors, most importantly phosphate, induce the osteo-/chondrogenic transdifferentiation of VSMCs via complex intracellular signaling pathways [3, 18, 29, 34, 54]. These cells are characterized by increased expression and activity of osteogenic transcription factors such as msh homeobox 2 (MSX2) [11] and core-binding factor α-1 (CBFA1) [9] as well as osteogenic enzymes such as tissue-nonspecific alkaline phosphatase (ALPL) [31, 43]. The osteoblast- and chondroblast-like VSMCs promote mineralization of vascular tissue via mechanisms similar to physiological bone calcification [39]. Inflammatory processes are involved in osteo-/chondrogenic transdifferentiation of VSMCs and vascular calcification in CKD [18, 46].

Increased plasma levels of interleukin-18 (IL-18), a pro-inflammatory cytokine [17], are frequently observed in CKD patients [12, 16] and associated with medial vascular calcification [23, 62]. Recent reports indicate that IL-18 enhances osteo-/chondrogenic transdifferentiation of VSMCs [62], but the intracellular signaling pathways contributing to these procalcific effects are still ill-defined.

The serum- and glucocorticoid-inducible kinase 1 (SGK1) is upregulated at transcriptional level by various pathologic factors known to trigger osteo-/chondrogenic transdifferentiation and calcification of VSMCs, such as hyperphosphatemia, mineralocorticoid or glucocorticoid excess, hyperglycemia, or inflammatory cytokines [25, 56–58]. SGK1 has been shown to contribute to the pathophysiology of several disorders [25, 30], including cardiac remodeling [53, 56, 57], hypertension [27], stroke [22], diabetes mellitus [26], kidney disease [10, 55], or inflammation [15]. SGK1 promotes osteo-/chondrogenic transdifferentiation of VSMCs [58]. Overexpression of constitutively active SGK1, but not inactive SGK1, is sufficient to induce osteo-/chondrogenic transdifferentiation of VSMCs [58]. Conversely, inhibition, deficiency, or knockdown of SGK1 are able to inhibit osteo-/chondrogenic transdifferentiation and calcification of VSMCs during phosphate exposure [58].

Therefore, the present study explored the effects of IL-18 on SGK1 expression in VSMCs as well as the possible involvement of SGK1 in IL-18-induced osteo-/chondrogenic transdifferentiation and calcification of VSMCs in vitro.

## Methods

### Cell culture of primary human aortic smooth muscle cells

Primary human aortic smooth muscle cells (HAoSMCs, Thermo Fisher Scientific) [2, 5, 34] were routinely cultured in medium containing Waymouth’s MB 752/1 medium and Ham’s F-12 nutrient mixture (Thermo Fisher Scientific) in a 1:1 ratio, supplemented with 10% FBS (Thermo Fisher Scientific), 100 U/ml penicillin, and 100 μg/ml streptomycin (Thermo Fisher Scientific). HAoSMCs were grown to confluence and used in all experiments from passages 4 to 10.

Where indicated, HAoSMCs were transfected with 10 nM SGK1 siRNA (ID no. s740, Thermo Fisher Scientific) or with 10 nM negative control siRNA (ID no. 4390843, Thermo Fisher Scientific) using siPORT amine transfection agent (Thermo Fisher Scientific) according to the manufacturer’s protocol [58]. The cells were used 48 h after transfection and silencing efficiency were analyzed by quantitative RT-PCR.

HAoSMCs were treated for 24 h (qRT-PCR), 7 days (ALPL activity), or 11 days (calcification) with 2 mM β-glycerophosphate (Sigma-Aldrich), the indicated concentrations of recombinant human interleukin-18 protein (R&D Systems), 50 μM SGK1 inhibitor EMD638683 (stock in DMSO) [57, 58], 1 μM LY294002 (Enzo Life Sciences, stock in DMSO), 100 nM wortmannin (Enzo Life Sciences, stock in DMSO) [21], or 1 μM PDK1 inhibitor GSK2334470 (Cayman Chemical, stock in DMSO). Equal amounts of vehicle were used as control. Treatment with calcification medium containing 10 mM β-glycerophosphate and 1.5 mM CaCl_2_ (Sigma-Aldrich) for 11 days was used for quantification of mineralization and Alizarin red staining [52, 59]. Fresh media with agents were added every 2–3 days.

### Quantification of calcium deposition

HAoSMCs were decalcified in 0.6 M HCl for 24 h at 4 °C. Calcium content in the supernatant was determined by using QuantiChrom Calcium assay kit (BioAssay Systems) according to the manufacturer’s protocol. HAoSMCs were lysed with 0.1 M NaOH/0.1% SDS and protein concentration was measured by the Bradford assay (Bio-Rad Laboratories). The results are shown normalized to total protein concentration [4].

### Alizarin red staining

To visualize calcification, HAoSMCs were fixed with 4% paraformaldehyde and stained with 2% Alizarin red (pH 4.5) [3]. The calcified areas are shown as red staining.

### ALPL activity assay

ALPL activity in HAoSMCs was determined by using the ALP colorimetric assay kit (Abcam) according to the manufacturer’s protocol. The results are shown normalized to total protein concentration measured by the Bradford assay (Bio-Rad Laboratories) [5, 54].

### Quantitative RT-PCR

Total RNA was isolated from HAoSMCs by using Trizol Reagent (Thermo Fisher Scientific) according to the manufacturer’s instructions [1, 33, 55]. Reverse transcription of 2 μg total RNA was performed using oligo(dT)_12–18_ primers (Thermo Fisher Scientific) and SuperScript III Reverse Transcriptase (Thermo Fisher Scientific). Quantitative RT-PCR was performed with the iCycler iQ™ Real-Time PCR Detection System (Bio-Rad Laboratories) and iQ™ Sybr Green Supermix (Bio-Rad Laboratories) according to the manufacturer’s instructions. The specificity of the PCR products was confirmed by analysis of the melting curves. All PCRs were performed in duplicate and relative mRNA expression was calculated by the 2^-ΔΔCt^ method using GAPDH as housekeeping gene, normalized to the control group. The following human primers were used (Thermo Fisher Scientific; 5′ → 3′ orientation):

*ALPL* fw: GGGACTGGTACTCAGACAACG;

*ALPL* rev: GTAGGCGATGTCCTTACAGCC;

*CBFA1* fw: GCCTTCCACTCTCAGTAAGAAGA;

*CBFA1* rev: GCCTGGGGTCTGAAAAAGGG;

*GAPDH* fw: GAGTCAACGGATTTGGTCGT;

*GAPDH* rev: GACAAGCTTCCCGTTCTCAG;

*MSX2* fw: TGCAGAGCGTGCAGAGTTC;

*MSX2* rev: GGCAGCATAGGTTTTGCAGC;

*SGK1* fw: GCAGAAGAAGTGTTCTATGCAGT;

*SGK1* rev: CCGCTCCGACATAATATGCTT.

### Western blotting

HAoSMCs were lysed with ice-cold IP lysis buffer (Thermo Fisher Scientific) containing complete protease and phosphatase inhibitor cocktail (Thermo Fisher Scientific) [45, 58]. After centrifugation at 10000 rpm for 5 min, protein concentrations were measured by the Bradford assay (Bio-Rad Laboratories). Equal amounts of proteins were boiled in Roti-Load1 Buffer (Carl Roth GmbH) at 100 °C for 10 min, separated on SDS-polyacrylamide gels and transferred to PVDF membranes. The membranes were incubated overnight at 4 °C with primary rabbit anti-SGK1 antibody (1:1000 dilution, cell signaling) or rabbit anti-GAPDH antibody (1:1000 dilution, cell signaling) and then with secondary anti-rabbit HRP-conjugated antibody (1:1000 dilution, cell signaling) for 1 h at room temperature. For loading controls, the membranes were stripped in stripping buffer (Thermo Fisher Scientific) at room temperature for 10 min. Antibody binding was detected with ECL detection reagent (Thermo Fisher Scientific), and bands were quantified by using ImageJ software. Results are shown as the ratio of total protein to GAPDH normalized to the control group.

### Statistics

Data are shown as scatter dot plots and arithmetic means ± SEM. *N* indicates the number of independent experiments performed at different passages of the cells. Normality was tested with Shapiro-Wilk test. Non-normal datasets were transformed (log) prior to statistical testing to provide normality according to Shapiro-Wilk test. Statistical testing was performed by one-way ANOVA followed by Tukey test for homoscedastic data or Games-Howell test for heteroscedastic data. Non-normal data were tested by the Steel-Dwass method. *P* < 0.05 was considered statistically significant.

## Results

**Fig. 1:**
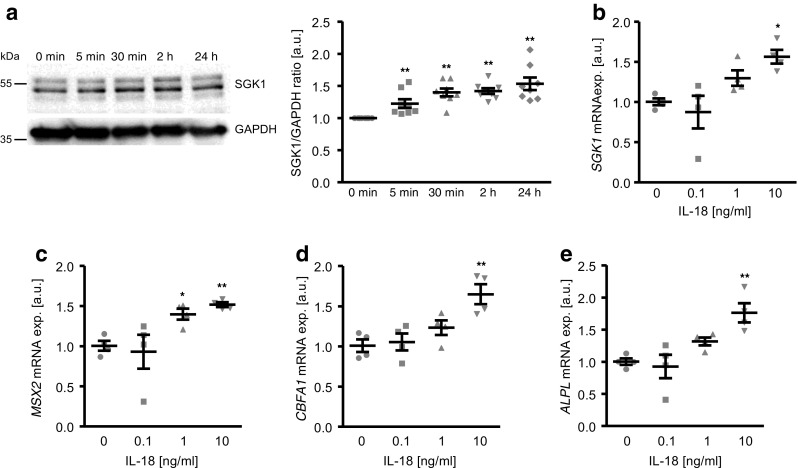
Interleukin-18 upregulates *SGK1* and osteogenic markers expression in primary human aortic smooth muscle cells in a dose-dependent manner. **a** Representative original Western blots and scatter dot plots and arithmetic means ± SEM (*n* = 8; arbitrary units, a.u.) of normalized SGK1/GAPDH protein ratio in HAoSMCs following treatment for the indicated times with 10 ng/ml recombinant human interleukin-18 protein (IL-18). **b–e** Scatter dot plots and arithmetic means ± SEM (*n* = 4; a.u.) of *SGK1* (**b**), *MSX2* (**c**), *CBFA1* (**d**), and *ALPL* (**e**) relative mRNA expression in HAoSMCs following treatment for 24 h with control (CTR) or with the indicated concentrations of recombinant human interleukin-18 protein (IL-18, 0.1–10 ng/ml). *(*p* < 0.05), **(*p* < 0.01) statistically significant vs. control-treated HAoSMCs

To investigate the effects of IL-18 on SGK1 expression in VSMCs, primary human aortic smooth muscle cells (HAoSMCs) were treated with recombinant human IL-18 protein. As shown in Fig. 1a, SGK1 protein abundance increased in HAoSMCs after 5 min and remained high up to 24 h following IL-18 treatment. IL-18 upregulated *SGK1* mRNA expression in HAoSMCs in a concentration-dependent manner (Fig. 1b). These effects reached statistical significance at 10 ng/ml IL-18 concentration.

Similarly, IL-18 treatment dose-dependently increased the mRNA expression of the osteogenic transcription factors *MSX2* and *CBFA1* (Fig. 1c, d) and of the osteogenic enzyme *ALPL* (Fig. 1e) in HAoSMCs, as markers of osteo-/chondrogenic transdifferentiation. Thus, the increased *SGK1* expression in IL-18 treated HAoSMCs was paralleled by increased osteo-/chondrogenic transdifferentiation.

**Fig. 2:**
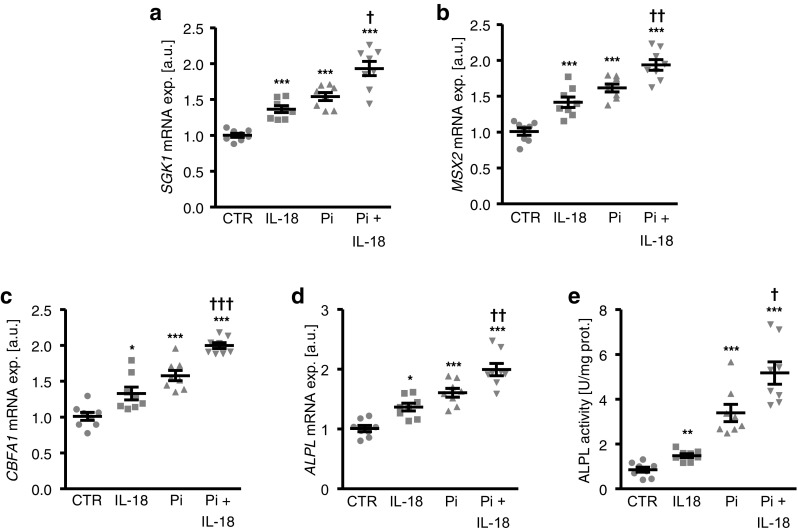
Interleukin-18 augments phosphate-induced *SGK1* expression and osteogenic signaling in primary human aortic smooth muscle cells. **a–d** Scatter dot plots and arithmetic means ± SEM (*n* = 8, arbitrary units, a.u.) of *SGK1* (**a**), *MSX2* (**b**), *CBFA1* (**c**), and *ALPL* (**d**) relative mRNA expression in HAoSMCs following treatment for 24 h with control or with 2 mM β-glycerophosphate (Pi) without or with additional treatment with 10 ng/ml recombinant human interleukin-18 protein (IL-18). **e** Scatter dot plots and arithmetic means ± SEM (*n* = 8, U/mg protein) of ALPL activity in HAoSMCs following treatment for 7 days with control or with 2 mM β-glycerophosphate (Pi) without or with additional treatment with 10 ng/ml recombinant human interleukin-18 protein (IL-18). *(*p* < 0.05), **(*p* < 0.01), ***(*p* < 0.001) statistically significant vs. control-treated HAoSMCs; †(*p* < 0.05), ††(*p* < 0.01), †††(*p* < 0.001) statistically significant vs. HAoSMCs treated with Pi alone

**Fig. 3:**
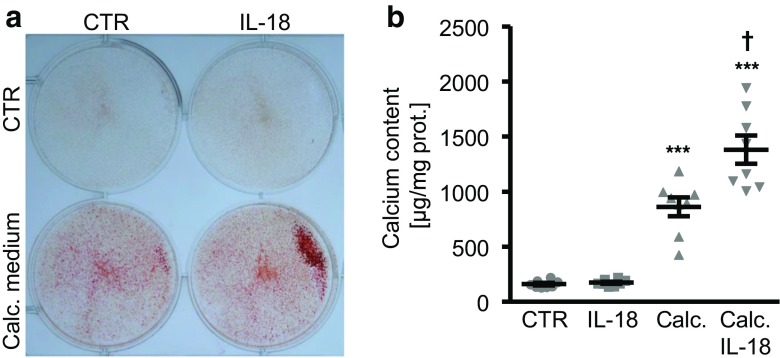
Interleukin-18 aggravates phosphate-induced calcification of primary human aortic smooth muscle cells. **a** Representative original images (*n* = 4) showing Alizarin red staining in HAoSMCs following treatment for 11 days with control or with calcification medium (calc. medium) without or with additional treatment with 10 ng/ml recombinant human interleukin-18 protein (IL-18). The calcified areas are shown as red staining. **b** Scatter dot plots and arithmetic means ± SEM (*n* = 8; μg/mg protein) of calcium content in HAoSMCs following treatment for 11 days with control or with calcification medium (calc.) without or with additional treatment with 10 ng/ml recombinant human interleukin-18 protein (IL-18). ***(*p* < 0.001) statistically significant vs. control-treated HAoSMCs; †(*p* < 0.05) statistically significant vs. HAoSMCs treated with calcification medium alone

Next, we explored the effects of IL-18 on *SGK1* expression and osteogenic signaling in HAoSMCs during high phosphate conditions. As shown in Fig. 2a, phosphate treatment upregulated *SGK1* mRNA expression in HAoSMCs, an effect significantly augmented by additional treatment with IL-18. Moreover, the phosphate-induced osteogenic markers *MSX2*, *CBFA1*, and *ALPL* mRNA expression (Fig. 2b–d) as well as ALPL activity (Fig. 2e) in HAoSMCs were significantly enhanced by IL-18 treatment. Alizarin red staining (Fig. 3a) and quantification of calcium deposition (Fig. 3b) in HAoSMCs revealed extensive calcification following treatment with calcification medium, effects again significantly aggravated by additional treatment with IL-18. Taken together, IL-18 augmented phosphate-induced *SGK1* expression, osteogenic signaling, and calcification of HAoSMCs.

**Fig. 4:**
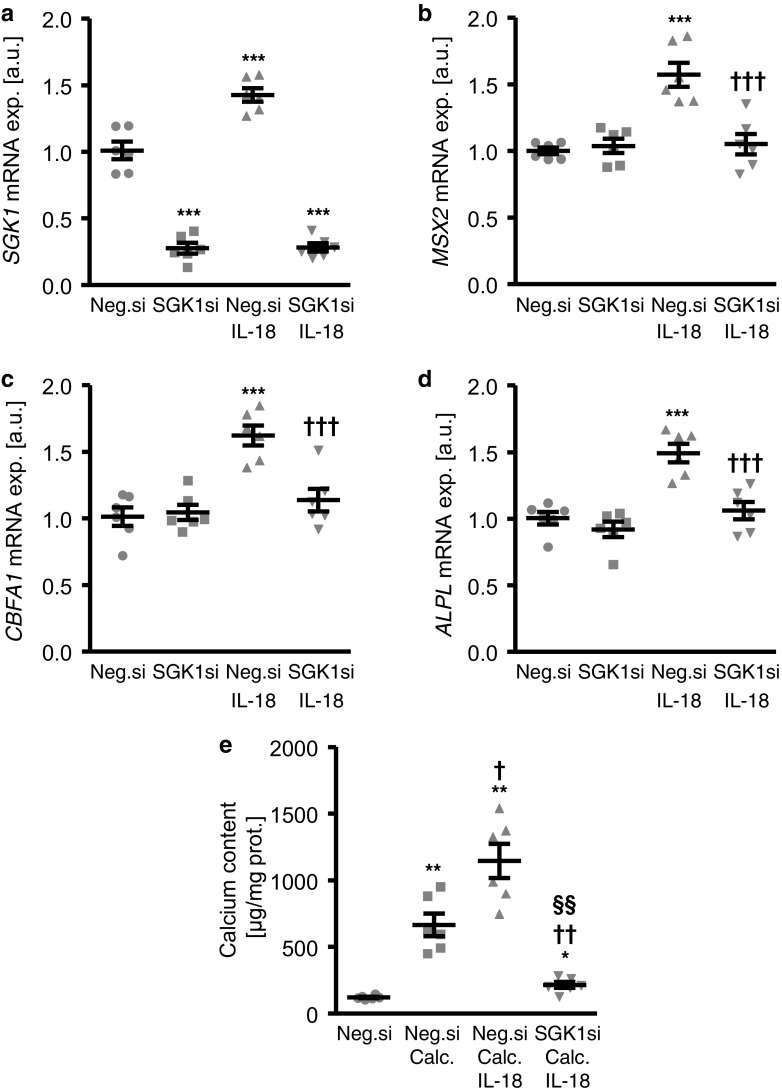
Silencing of SGK1 blunts interleukin-18-induced osteogenic markers expression and calcification of primary human aortic smooth muscle cells. **a–d** Scatter dot plots and arithmetic means ± SEM (*n* = 6, arbitrary units, a.u.) of *SGK1* (**a**), *MSX2* (**b**), *CBFA1* (**c**), and *ALPL* (**d**) relative mRNA expression in HAoSMCs following silencing for 48 h with negative control siRNA (neg.si) or SGK1 siRNA (SGK1si) without or with additional treatment for 24 h with 10 ng/ml recombinant human interleukin-18 protein (IL-18). ***(*p* < 0.001) statistically significant vs. neg.si-silenced HAoSMCs; †††(*p* < 0.001) statistically significant vs. neg.si-silenced and IL-18-treated HAoSMCs. **e** Scatter dot plots and arithmetic means ± SEM (*n* = 6; μg/mg protein) of calcium content in HAoSMCs following 11 days of silencing with negative control siRNA (neg.si) or SGK1 siRNA (SGK1si) and additional treatment with control or with calcification medium (calc.) without or with additional treatment with 10 ng/ml recombinant human interleukin-18 protein (IL-18). *(*p* < 0.05), **(*p* < 0.01) statistically significant vs. neg.si-silenced HAoSMCs; †(*p* < 0.05), ††(*p* < 0.01) statistically significant vs. neg.si-silenced and calcification medium alone-treated HAoSMCs; §§(*p* < 0.01) statistically significant vs. neg.si-silenced and calcification medium with IL-18-treated HAoSMCs

To investigate the possible involvement of SGK1 in IL-18-induced osteo-/chondrogenic transdifferentiation of VSMCs, the endogenous expression of SGK1 was suppressed by silencing of the *SGK1* gene in HAoSMCs followed by additional treatment without or with IL-18. As a result, *SGK1* mRNA expression was significantly lower in SGK1 siRNA transfected HAoSMCs as compared to negative control siRNA silenced HAoSMCs (Fig. 4a). IL-18 treatment upregulated *SGK1* mRNA expression in negative control silenced HAoSMCs. The IL-18-induced mRNA expression of *MSX2*, *CBFA1*, and *ALPL* in negative control silenced HAoSMCs was significantly blunted in SGK1 silenced HAoSMCs (Fig. 4b–d). Furthermore, the augmentation of HAoSMCs calcification by IL-18 in the presence of calcification medium was reversed by SGK1 knockdown (Fig. 4e). In accordance with the previous observations showing protective effects of SGK1 inhibition during high phosphate conditions, silencing of SGK1 significantly inhibited calcium deposition in HAoSMCs beyond counteracting the procalcific effects of IL-18. Taken together, the procalcific effects of IL-18 in HAoSMCs were, at least in part, dependent on SGK1.

**Fig. 5:**
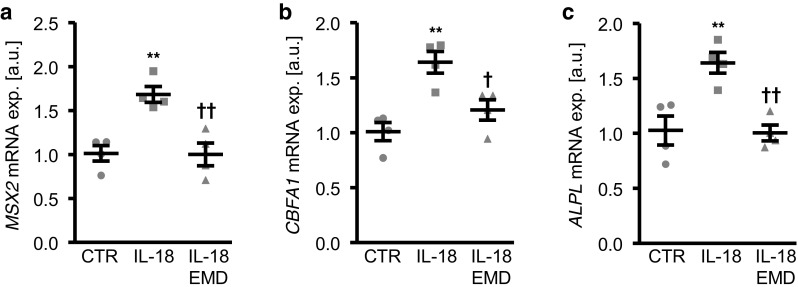
SGK1 inhibition suppresses interleukin-18-induced osteogenic markers expression in primary human aortic smooth muscle cells. **a–c** Scatter dot plots and arithmetic means ± SEM (*n* = 4, a.u.) of *MSX2* (**a**), *CBFA1* (**b**), and *ALPL* (**c**) relative mRNA expression in HAoSMCs following treatment for 24 h with control (CTR) or with 10 ng/ml recombinant human interleukin-18 protein (IL-18) without or with additional treatment with 50 μM SGK1 inhibitor EMD638683 (EMD). **(*p* < 0.01) statistically significant vs. control-treated HAoSMCs; †(*p* < 0.05), ††(*p* < 0.01) statistically significant vs. IL-18 alone-treated HAoSMCs

The involvement of SGK1 in IL-18-induced osteo-/chondrogenic transdifferentiation of VSMCs was confirmed by treatment of HAoSMCs with IL-18 in the presence or absence of the SGK1 specific inhibitor EMD638683. As shown in Fig. 5a–c, SGK1 inhibitor EMD638683 similarly suppressed the IL-18-induced upregulation of *MSX2*, *CBFA1*, and *ALPL* mRNA expression in HAoSMCs.

**Fig. 6:**
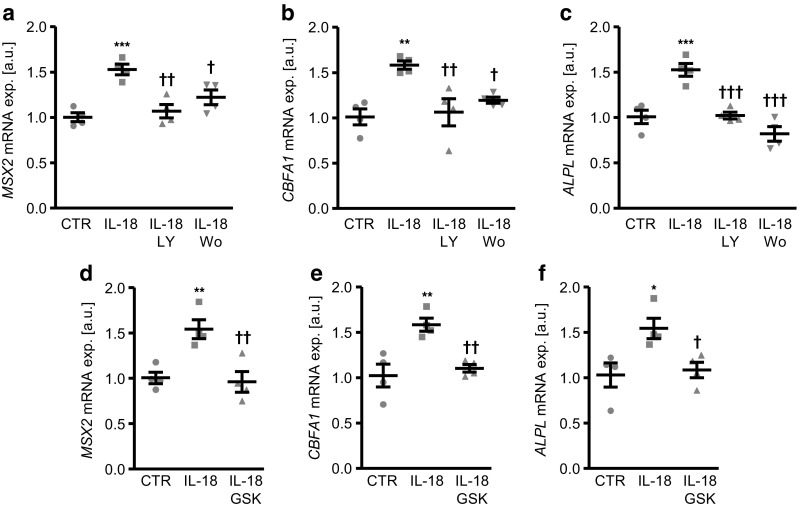
Interleukin-18-induced osteo-/chondrogenic markers expression is suppressed by inhibition of PI3K pathway in primary human aortic smooth muscle cells. **a** Scatter dot plots and arithmetic means ± SEM (*n* = 4; a.u.) of *MSX2* (**a**), *CBFA1* (**b**), and *ALPL* (**c**) relative mRNA expression in HAoSMCs following treatment for 24 h with control (CTR) or with 10 ng/ml recombinant human interleukin-18 protein (IL-18) without or with additional treatment with the PI3K inhibitors 1 μM LY294002 (LY) or 100 nM wortmannin (Wo). Scatter dot plots and arithmetic means ± SEM (*n* = 4; a.u.) of *MSX2* (**d**), *CBFA1* (**e**), and *ALPL* (**f**) relative mRNA expression in HAoSMCs following treatment for 24 h with control (CTR) or with 10 ng/ml recombinant human interleukin-18 protein (IL-18) without or with additional treatment with 1 μM PDK1 inhibitor GSK2334470 (GSK). *(*p* < 0.05), **(*p* < 0.01), ***(*p* < 0.001) statistically significant vs. control-treated HAoSMCs; †(*p* < 0.05), ††(*p* < 0.01), †††(*p* < 0.001) statistically significant vs. HAoSMCs treated with IL-18 alone

**Fig. 7:**
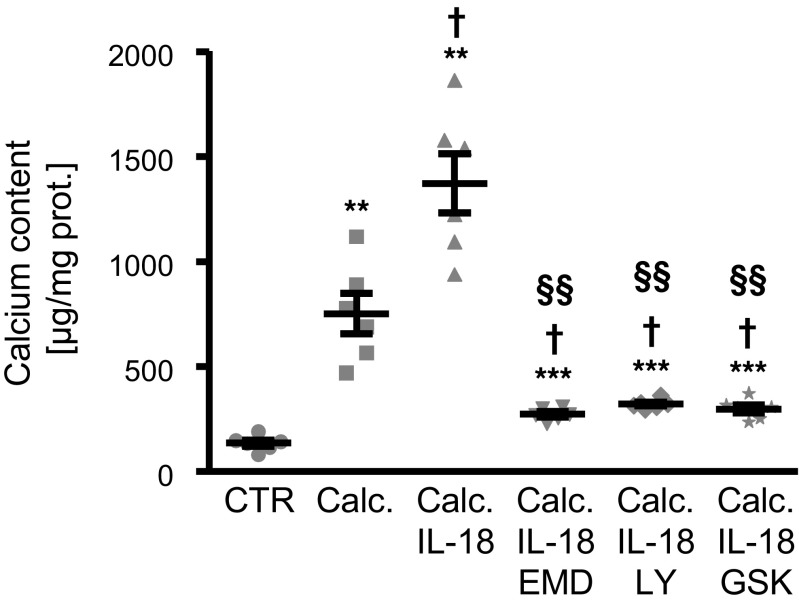
Inhibition of SGK1 or PI3K pathway blunt the effects of interleukin-18 on calcification of primary human aortic smooth muscle cells. Scatter dot plots and arithmetic means ± SEM (*n* = 6; μg/mg protein) of calcium content in HAoSMCs following treatment for 11 days with control or with calcification medium (calc.) without or with additional treatment with 10 ng/ml recombinant human interleukin-18 protein (IL-18) alone or together with 50 μM SGK1 inhibitor EMD638683 (EMD), 1 μM PI3K inhibitor LY294002 (LY), or 1 μM PDK1 inhibitor GSK2334470 (GSK). **(*p* < 0.01), ***(*p* < 0.001) statistically significant vs. control-treated HAoSMCs; †(*p* < 0.05) statistically significant vs. calcification medium alone-treated HAoSMCs; §§(*p* < 0.01) statistically significant vs. calcification medium with IL-18 alone-treated HAoSMCs

Additional experiments were performed to elucidate the regulation of SGK1-dependent osteogenic signaling in IL-18 treated HAoSMCs. To this end, the roles of phosphatidylinositol-4,5-bisphosphate 3 kinase **(**PI3K) and 3-phosphoinositide-dependent protein kinase 1 (PDK1), critical upstream kinases in SGK1 regulation were investigated. As shown in Fig. 6a–c, the upregulation of *MSX2*, *CBFA1*, and *ALPL* mRNA expression following treatment with IL-18 was significantly inhibited in the presence of either PI3K inhibitors LY294002 or wortmannin. In addition, IL-18-induced osteogenic markers mRNA expression was suppressed by additional treatment with the PDK1 inhibitor GSK2334470 (Fig. 6d–f). Thus, IL-18-induced osteo-/chondrogenic signaling in VSMCs involved PI3K pathway activation. Accordingly, pharmacological inhibition of SGK1, PI3K, or PDK1, all suppressed calcium deposition in HAoSMCs triggered by calcification medium supplemented with IL-18 (Fig. 7).

## Discussion

**Fig. 8:**
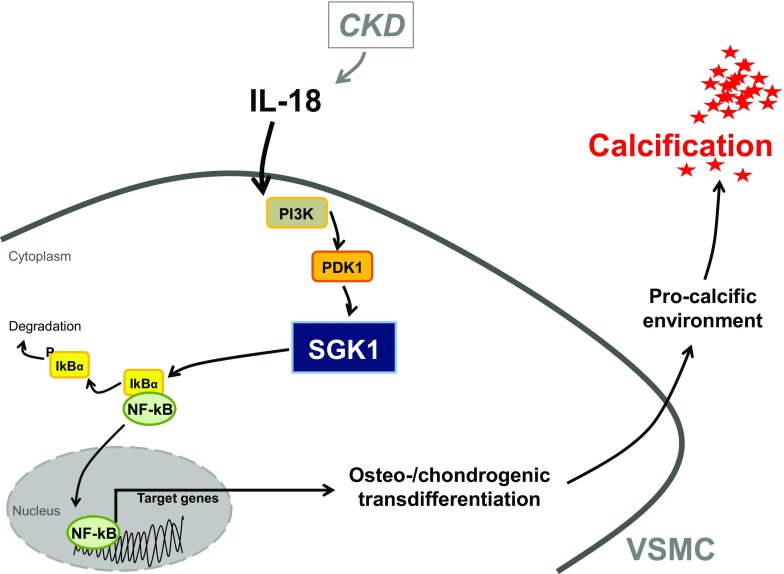
Schematic illustration of SGK1-dependent IL-18-induced VSMC calcification. CKD increases circulating IL-18 levels [12, 16]. In VSMCs, IL-18 may activate PI3K/PDK1 signaling to increase the expression and activity of SGK1. Further, SGK1 may induce transcriptional activation of NF-kB via phosphorylation-dependent ubiquitination and degradation of IkBα [25] and, thus, the expression of NF-kB target genes to promote osteo-/chondrogenic transdifferentiation of VSMCs [58]. The osteo-/chondrogenic transdifferentiation of VSMCs induces a procalcific environment causing vascular calcification (CKD, chronic kidney disease; IL-18, interleukin 18; PI3K, phosphatidylinositol-4,5-bisphosphate 3 kinase; PDK1, 3-phosphoinositide-dependent protein kinase 1; SGK1, serum- and glucocorticoid-inducible kinase 1; NF-kB, nuclear factor “kappa-light-chain-enhancer” of activated B-cells; IkBα, nuclear factor of kappa light polypeptide gene enhancer in B cells inhibitor, alpha)

The present study discloses a novel key role of SGK1 in the signaling of IL-18-induced osteo-/chondrogenic transdifferentiation of VSMCs in-vitro. IL-18 upregulates *SGK1* mRNA expression in VSMCs while silencing or pharmacological inhibition of SGK1 suppresses IL-18-induced osteogenic markers expression, indicative of increased osteo-/chondrogenic transdifferentiation (Fig. 8).

IL-18, a pro-inflammatory cytokine from the IL-1 cytokine family [17], is produced by various cells including osteoblasts [47], chondrocytes [38], and VSMCs [17]. IL-18 induces cellular inflammatory responses by binding to the IL-18 receptor [37]. VSMCs express a functional IL-18 receptor [17, 48]. Conversely, inflammatory processes upregulate IL-18 expression [14]. Increased plasma IL-18 levels are frequently observed in CKD patients [12, 16, 62]. IL-18 is involved in the progression of CKD [46, 51] and its related complications including vascular calcification [46, 62]. Similar to previous reports [62], our observations show that IL-18 treatment enhances phosphate-induced osteo-/chondrogenic transdifferentiation and calcification of VSMCs. In addition, IL-18 treatment alone is able to induce the expression of osteogenic markers in primary human aortic VSMCs.

The procalcific effects of IL-18 are mediated, at least in part, by SGK1. IL-18 upregulates SGK1 expression and augments phosphate-induced SGK1 expression in VSMCs. Excessive SGK1 is sufficient to promote osteo-/chondrogenic transdifferentiation of VSMCs via NF-kB activation [58] and NF-kB activity can be induced by IL-18 [24, 42, 50, 60]. SGK1 similarly plays a key role in vascular inflammation during atherogenesis via activation of NF-kB [7]. We could show here that silencing or pharmacological inhibition of SGK1 in VSMCs is able to suppress IL-18-induced osteogenic markers expression and calcium deposition, suggesting that SGK1 is a key factor in the signaling leading to osteo-/chondrogenic transdifferentiation and calcification of VSMCs during conditions of high IL-18 levels.

Moreover, IL-18 is able to activate PI3K-dependent signaling in various cell types [8, 61, 63] including VSMCs [40], while PI3K activation leads to increased expression and activity of SGK1 [6, 13, 19, 25, 30]. Accordingly, PI3K and PDK1 inhibition suppresses IL-18-dependent osteoinduction in VSMCs.

The present observations identify SGK1 regulation as the key mechanism of IL-18-induced osteo-/chondrogenic transdifferentiation of VSMCs. However, other effects may contribute to the procalcific effects of IL-18 in VSMCs. TRPM7 is a ubiquitously expressed Mg^2+^-permeable ion channel [49] with a complex role during vascular calcification [36], which has been suggested to contribute to the effects of IL-18 [62]. SGK1 is able to enhance TRPM7 expression [49]. Thus, TRPM7 expression may also be a downstream product in the SGK1-dependent signaling during calcifying conditions.

Taken together, SGK1 appears critically important in the progression of vascular calcification during inflammatory conditions with high IL-18 levels such as CKD [12, 16, 62]. In addition, SGK1 mediates the induction of osteo-/chondrogenic transdifferentiation and, thus, vascular calcification by other pathological factors such as hyperphosphatemia or mineralocorticoid excess [58]. Thus, SGK1 may be a central key regulator in the signaling pathways mediating vascular calcification during various pathological conditions, including inflammation. Therefore, SGK1 inhibition may be a feasible therapeutic target to reduce the progression of vascular calcification triggered by complex conditions such as CKD [28, 29, 35, 44]. Moreover, SGK1 inhibition may potentially have an overall protective effect during cardiovascular or renal disease progression [10, 25, 27, 30, 55].

In conclusion, IL-18 upregulates SGK1 expression in VSMCs and SGK1 participates in the intracellular signaling mediating the IL-18-induced osteo-/chondrogenic transdifferentiation of VSMCs. Thus, SGK1 inhibition may be beneficial in reducing the progression of medial vascular calcification during vascular inflammatory conditions such as CKD.
